# More Older Adults Try Patient Portals in Recent Years, but Many Don’t Continue: A Meta-Analysis

**DOI:** 10.1145/3772363.3798366

**Published:** 2026-04-13

**Authors:** Nina Sakhnini, Debaleena Chattopadhyay

**Affiliations:** Computer Science, University of Illinois at Chicago, Chicago, Illinois, USA; Computer Science, University of Illinois Chicago, Chicago, Illinois, USA

**Keywords:** older adults, patient portals, meta-analysis, continued use

## Abstract

Patient portals are a convenient way for patients to communicate with healthcare providers and manage care. Active portal engagement is associated with enhanced knowledge, increased self-efficacy, and improved symptom management. However, gaps in portal use persist, particularly among older adults. We conducted a random-effects meta-analysis of studies published between 2005 and 2025, with publication year as a moderator. From 1,487 unique records, 18 studies were included in the meta-analysis. We found that an estimated 55% of older adults have used a patient portal at least once, and 49% were continuing users, although these rates varied widely across studies. Publication year was a significant moderator for ever having used a patient portal, suggesting increasing uptake over time, but was not significantly associated with continued use. These findings suggest that improving onboarding alone may not be sufficient; ongoing tech support is needed to sustain long-term patient portal engagement among older adults.

## 1 Introduction

Patient-facing digital health tools are now embedded in healthcare systems worldwide. For example, patient portals have become a common mechanism for delivering timely information and supporting self-management across diverse care settings [2, 28]. Portals support everyday tasks such as accessing test results, requesting prescription refills, scheduling appointments, and communicating with clinicians [40]. However, portal uptake remains uneven: research consistently finds lower use among people with low income, lower education or literacy, limited English proficiency, and older age [13, 32]. When these groups cannot easily access or use portals, digital health workflows exacerbate disparities and raise costs for both patients and health systems [26].

In the U.S., public policy has further accelerated the availability of portal-based access to electronic health information (EHI). The 21st Century Cures Act emphasizes secure, patient-authorized access to EHI, and most U.S. healthcare organizations now offer portal-based services [39]. The COVID-19 pandemic also increased interest in and use of patient portals for services such as telehealth [32]. However, in 2024, 77% of Americans reported being offered access to their online medical records, but only 65% reported actually accessing them [40]. This gap raises ongoing concerns about “techquity” in portal uptake and use [40, 42], with barriers ranging from low technology literacy and limited English proficiency to poor usability and lack of technical support. Among the populations for whom portal use is strongly advocated but remains problematic are older adults [48]. Compared to the general population, older adults have greater healthcare needs [19], including more frequent hospitalizations [1], comorbidities [41], and multiple chronic conditions [49]. Historically, portal uptake and use have been low among older adults [51]. In the last five years, efforts to increase portal use have expanded substantially, including dedicated staff to support uptake [30], mobile apps that broaden access [25], and authentication that improves security [11]. Yet it remains unclear whether increased access and onboarding efforts translate into sustained portal use among older adults.

In this paper, we synthesize evidence on patient portal use among older adults over the last two decades. Models of older adults’ technology acceptance and learning suggest that trying a tool and integrating it into routine practice are two distinct milestones; trial and experimentation only sometimes lead to sustained use, depending on factors such as perceived utility and access to technical support [4, 38, 44]. Because many patient portal studies measure behavior rather than intention, we focus on two behavioral stages: *ever-use* (trial use; using a patient portal at least once) and *continued use* (ongoing engagement over time). Conflating these stages can obscure where breakdowns occur—whether barriers primarily prevent older adults from getting started, or whether recurring friction (e.g., authentication hurdles, navigation complexity, and comprehension demands) undermines sustained engagement. We systematically reviewed empirical studies of older adults’ patient portal use and conducted random-effects meta-analyses to synthesize evidence on ever-use and continued use. This work provides pooled prevalence estimates for both stages of use and motivates design and support implications that extend beyond onboarding to sustaining patient portal engagement for older adults.

## 2 Methods

We conducted a random-effects meta-analysis [5] to pool study-level prevalence estimates and account for between-study heterogeneity. We then used meta-regression with publication year to test whether ever-use and continued use differed over time. Following standard systematic review and meta-analysis procedures, we developed a comprehensive search strategy, applied predefined inclusion and exclusion criteria, extracted study-level data, and synthesized results from eligible studies. In parallel, we conducted a qualitative thematic analysis to contextualize the quantitative findings and identify recurring barriers and facilitators shaping older adults’ portal engagement [6, 7].

We developed search strings combining terms for *patient portals* (e.g., patient portal, eHealth), *caregivers* (e.g., informal caregiver, family caregiver, and spousal caregiver), *alzheimer’s disease and related dementia* (e.g., people with dementia, diagnosed with MCI, and PwD), *older adults* (e.g., older adult, elderly, seniors), and *method* (e.g., user study, interview, participatory design) incorporating database-specific controlled vocabulary where available. ADRD terms were added to the search strategy to avoid undersampling of the literature, since older adults are sometimes identified by diagnosis rather than age group in health informatics studies. Full search terms are provided in Appendix A. We ran the searches in August 2025 across nine databases (ACM Digital Library, PubMed, Web of Science, Scopus, ERIC, Embase, PsycInfo, EBSCO CINAHL, Chocrane) and supplemented results with citation chaining. We limited results to publications from January 2005 through July 2025. We followed the Preferred Reporting Items for Systematic Reviews and Meta-Analyses (PRISMA) framework to document screening and inclusion decisions [18] (see Figure 4 in Appendix A).

Studies were included if they (1) reported an empirical finding, (2) included older adults (as defined by each study), and (3) provided extractable quantitative data to calculate the prevalence of patient portal engagement (e.g., number of portal users and total sample size). We excluded studies that did not report sufficient quantitative data, focused exclusively on clinicians, or evaluated non-portal technologies. After deduplication, records were screened in two stages: title and abstract screening followed by full-text review. The first author assessed the relevance of search results, selected full texts for further review, and extracted key study characteristics using a standardized data extraction form. The first and second authors jointly determined study eligibility, resolving discrepancies through discussion; when disagreement persisted, the second author made the final decision. Using these criteria, we included 18 studies (18 articles) in the meta-analysis from a total of 1,487 unique records (see Figure 4 in Appendix A).

For each included study, we extracted study characteristics (e.g., setting, measures, and definition of an older adult) and quantitative data needed to estimate prevalence. During extraction, we distinguished between studies that operationalized portal engagement as ever-use (trial use; used a portal at least once) and continued use (ongoing use beyond onboarding, as defined by each study mainly as repeated portal access or sustained activity over a specified observation period, e.g., multiple logins, active use across months, or classification as an ‘active user’ based on a defined criteria). Because these operationalizations reflect distinct engagement stages and some studies used overlapping samples with different definitions, we conducted subgroup analyses and did not compute a single pooled estimate across both outcomes. Effect sizes were computed as logit-transformed proportions from the number of users and total sample size; models were fit using restricted maximum likelihood (REML). We report pooled prevalence with 95% confidence intervals and heterogeneity statistics (*τ*^2^, *I*^2^, *Q*).

To examine temporal patterns, we fit mixed-effects meta-regression models with publication year as a continuous moderator. Publication year was used because data-collection years were inconsistently reported, and it provides a consistent temporal proxy. As a complementary analysis of temporal direction using aggregated counts, we additionally fit a binomial logistic regression weighted by study sample size; because this approach can be sensitive to influential large studies, we interpret it as supportive rather than primary evidence. We assessed risk of bias using the Joanna Briggs Institute (JBI) Critical Appraisal Checklist for Prevalence Studies [31]. The first and second authors independently assessed each study and resolved disagreements through discussion. We retained all eligible studies and interpreted findings in light of the overall risk profile. In parallel, we conducted a reflexive thematic analysis [6, 7] to contextualize prevalence estimates and identify recurring factors shaping older adults’ portal engagement, focusing on usability and design barriers, access to technical support, and caregiver involvement. We extracted qualitative findings and developed open codes capturing factors shaping portal engagement. Through iterative axial coding and team discussion, codes were grouped into higher-level themes and finalized by consensus.

## 3 Results

### 3.1 Meta-Analysis

Among the 18 studies included in the meta-analysis, nine reported the prevalence of older adults who had ever used a patient portal [3, 9, 10, 16, 17, 22, 34, 37, 50], and nine reported continued use [8, 12, 14, 20, 23, 29, 36, 46, 47]. Eleven studies defined older adults as age 65+ [8–10, 12, 14, 16, 17, 20, 29, 34, 36] while seven as 50+ [3, 22, 23, 37, 46, 47, 50]. Publication years ranged from 2014 to 2025, and studies spanned three countries (U.S., Netherlands, and Argentina), with the majority conducted in the U.S. (15/18) [3, 8–10, 12, 16, 17, 20, 22, 23, 34, 36, 37, 46, 47]. We observed two study contexts: (1) health-system–based studies that focused on a single institution’s portal, and (2) community/population studies that sampled older adults across multiple health organizations (and thus multiple portals [3, 8, 9, 12, 20, 22, 46, 47]). Health-system–based studies examined single-portal implementations within large health systems (e.g., Ochsner [37], Atrium [10, 23], AMC Amsterdam [50], Kaiser Permanente [16, 17, 36], HIBA [14]), most commonly Epic MyChart, with a few bespoke portals [14, 29]. Data collection also varied: studies used national [8] or statewide surveys [12], systemwide institutional surveys [14, 16, 17, 23, 29, 34, 36, 37, 50], and study-specific community instruments [3, 9, 20, 22, 46, 47]. Some institutional studies supplemented surveys with portal usage logs [16, 17, 50] (one study used logs only [10]); all log-based studies were in the ever-use group.

Across nine studies [3, 9, 10, 16, 17, 22, 34, 37, 50], the pooled prevalence of older adults who had *ever used* a patient portal was 0.55 (95% CI: 0.28–0.79). Heterogeneity was very high, *τ*^2^ = 2.93, *I*^2^ = 100%, *Q*(8) = 234,212.58, *p* < .0001. Publication year was a significant moderator in mixed-effects meta-regression, *QM* (1) = 7.27, *p* = 0.007. The minimum inclusion age was also a significant moderator, *QM*(1) = 4.99, *p* = 0.026. Reporting method (self-report, logs, or both) and study country were not significant moderators.

Across the other nine studies [8, 12, 14, 20, 23, 29, 36, 46, 47], the pooled prevalence of *continued* patient portal *use* was 0.49 (95% CI: 0.31–0.67). Heterogeneity remained very high but was lower than in the ever-use subgroup, *τ*^2^ = 1.25, *I*^2^ = 97.6%, *Q*(8) = 550.55, *p* < .0001. Publication year was not a significant moderator, *QM*(1) = 0.35, *p* = 0.55. The minimum inclusion age and study country were not significant moderators. Reporting method was not tested as a moderator because all included studies relied on self-report.

**Figure 3:**
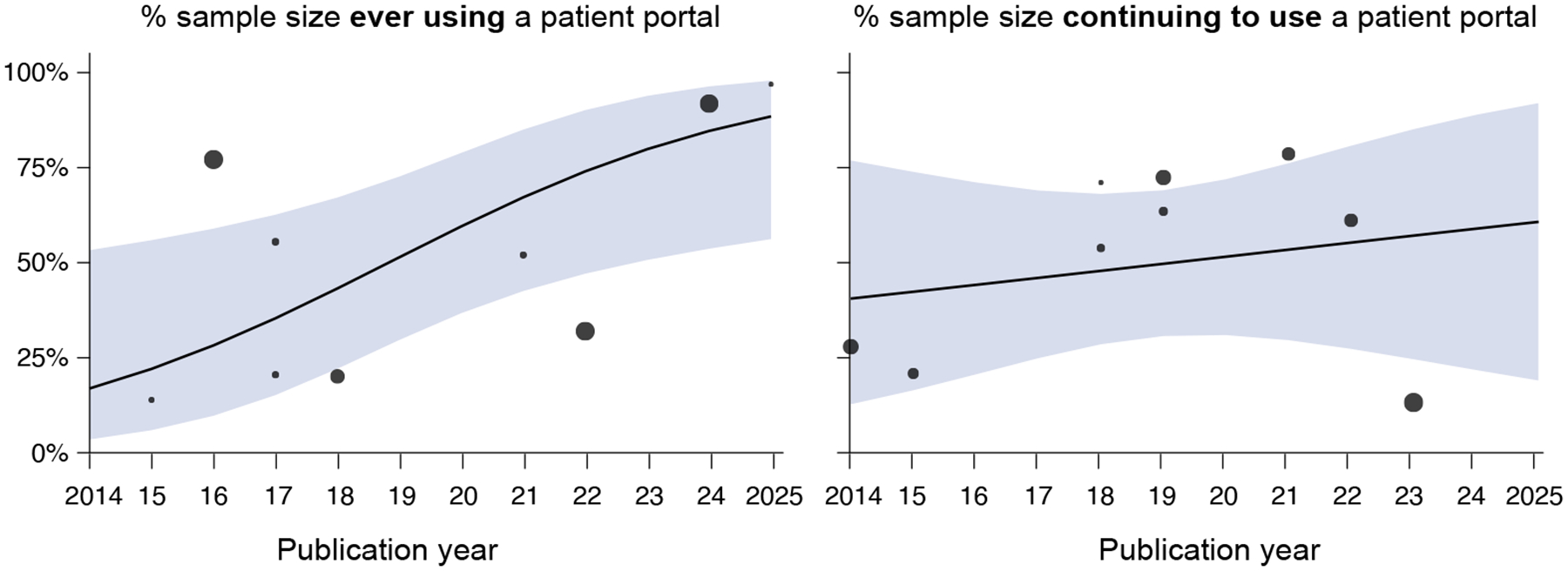
Meta-regression of patient portal engagement by publication year. Publication year significantly moderated the prevalence of older adults who have used a patient portal (at least once), *QM* (1) = 7.27, *p* = 0.007; OR/year = 1.39, 95% CI 1.06–1.83, *p* = 0.018 (left), but was not a significant moderator of continued use, *QM*(1) = 0.35, *p* = 0.55 (right). Thus, the odds of older adults ever using a patient portal (ever-use) appeared about 39% higher for each more recent year.

**Figure 4:**
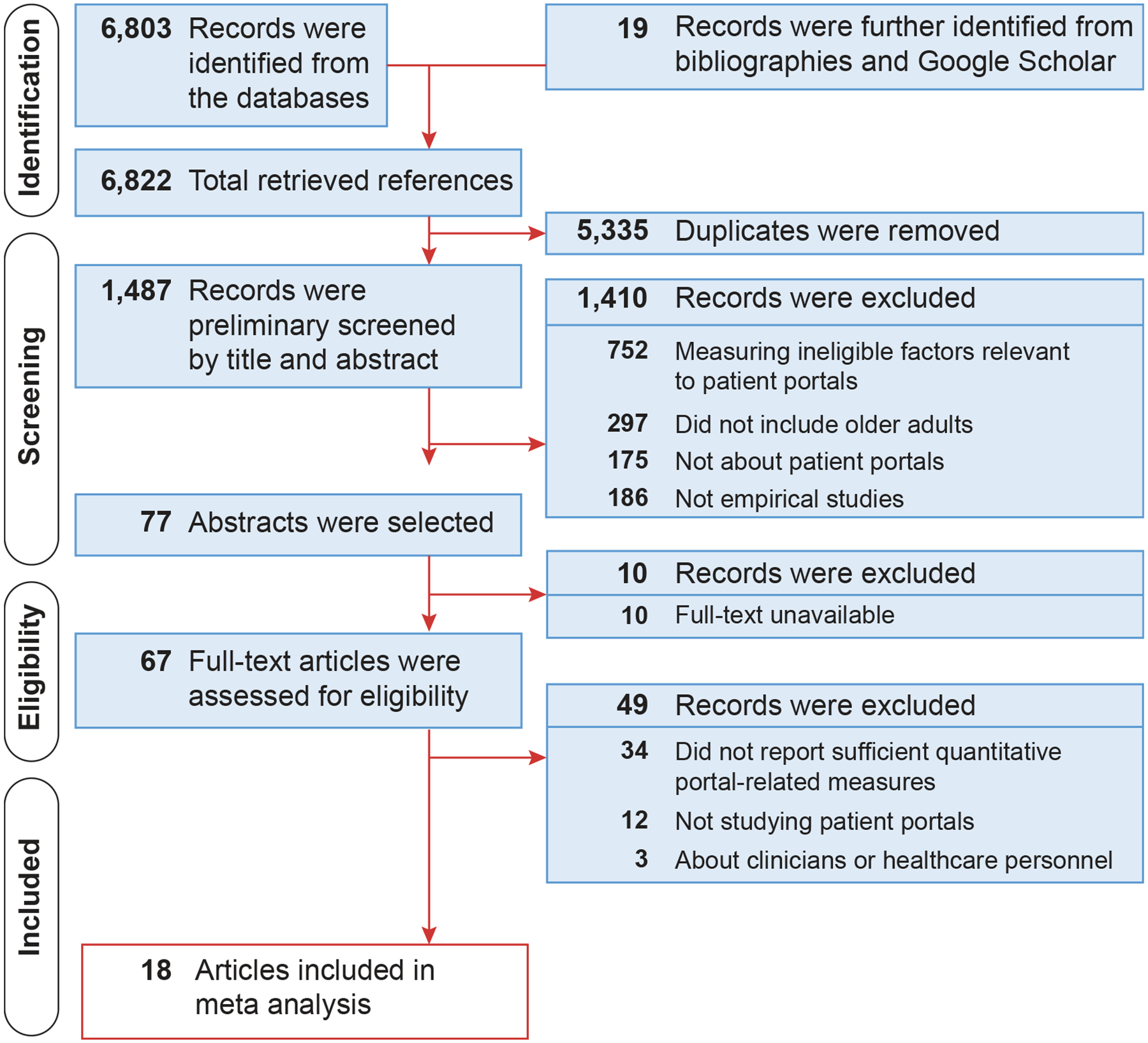
Summary of literature search following PRISMA guidelines [35].

To further characterize temporal patterns across engagement stages, we examined publication year as a moderator using mixed-effects meta-regression. Meta-regression found publication year significantly moderated ever-use, OR/year = 1.39, 95% CI 1.06–1.83, *p* = 0.018, suggesting that studies published in more recent years reported higher odds of older adults ever using a patient portal (about 39% higher per additional publication year; Figure 3). In supplementary analyses using sample-size–weighted logistic regression on aggregated counts, ever-use also increased over time (OR/year = 1.11, i.e., 11.1% higher odds per year; Appendix Figure 5). Continued use showed the opposite pattern in the logistic model (OR/year = 0.89, i.e., 11% lower odds per year; Appendix Figure 5). Because these count-based analyses can be sensitive to influential large studies, we interpret them as complementary to the meta-regression results.

Overall, the risk of bias was frequently very high (Appendix B). Of the 18 studies, 12 were rated High risk, five Moderate, and one Low. High risk was more common in continued-use studies (7/9) than in ever-use studies (5/9), primarily due to non-representative samples, reliance on self-reported use, and limited response-rate reporting.

### 3.2 Qualitative analysis

Across the included studies, older adults’ portal engagement was shaped by a combination of interface-level friction, perceived value, and structural support. Usability challenges were repeatedly described as barriers not only to getting started but also to continued engagement. Older adults reported difficulty with authentication (e.g., repeated password entry, multi-step login flows, and two-factor authentication), readability and targeting issues due to small fonts/icons and low-contrast design, and non-intuitive navigation that made it hard to locate key functions or complete tasks [3, 16, 20, 36, 37, 46, 50]. Portals also frequently relied on medical and technical terminology that increased interpretation burden and frustrated users [20, 36, 37, 50]. Consistent with these challenges, one study reported low perceived usability (mean 28.7/40) [46]. Across studies, limited availability of multimodal training and ongoing technical support further constrained continued use, particularly when problems arose during real-world tasks [16, 37, 46].

At the same time, studies highlighted clear motivations for initial uptake. Ever-use was more common among older adults with higher education, greater health and eHealth literacy, prior or regular internet use, and being married [3, 10, 16, 17, 22, 37, 50]. Older adults valued portals most for accessing personal health information—especially laboratory results and medical records—and for timely communication with clinicians, along with convenience features such as scheduling and prescription refills [20, 34, 46, 47, 50]. Several studies also described empowerment and autonomy in health decision-making as motivators of engagement [34, 46, 47].

However, structural and social barriers continued to shape who benefits from portals [3, 8, 9, 12, 14, 16, 17, 36, 37, 46, 47, 50]. Across studies, adults aged 70+ consistently had lower portal use [16, 17, 36, 37, 50]. Studies also documented racial and ethnic inequities, including lower activation and feature use among African American, Latino, Filipino, and other groups [3, 16, 17]. Lower income, education, and digital/health literacy were also associated with reduced portal use and engagement [3, 8, 9, 12, 14, 22, 37, 46, 47]. Limited device access, lack of home internet, and the cost of connectivity were recurring barriers [3, 16, 17, 22]. Even when access was available, limited technology proficiency and insufficient technical support remained common constraints [22, 36, 37]. Provider-side factors also mattered: delayed or absent responses to portal messages reduced satisfaction and undermined confidence in portal value [12, 29, 50].

Finally, caregiver involvement often enabled older adults’ portal use, but in complex ways. Caregivers often accessed portals by sharing login credentials rather than using formal proxy mechanisms, with spousal caregivers more likely to engage than adult children or other relatives [8, 9, 23]. Caregivers viewed portal access as important for coordinating care and supporting communication, especially for older adults with cognitive impairments [9, 23, 50]. Yet older adults were often unaware of proxy features and raised concerns about privacy and autonomy when access occurred without granular permission controls. Informal proxy use also complicates the interpretation of usage logs because caregiver activity is indistinguishable from patient activity [8, 10, 23].

## 4 Discussion

Across included studies, an estimated 55% of older adults had ever used a patient portal, while about 49% were continued users. Ever-use appeared to increase across publication years (OR/year = 1.39, about 39% higher odds per year), but continued use did not point to a comparable increase. Our synthesis suggests that, year over year, more older adults may be trying patient portals at least once, which may reflect an increasing effectiveness of onboarding-focused institutional initiatives in “getting people in the door.” This divergence is consistent with a drop-off after first use—older adults may be trying portals more often, but many may not be transitioning from trial to sustained engagement.

This pattern aligns well with frameworks of older adults’ technology adoption that distinguish early trial and experimentation from later acceptance and routine use [4, 38]. Technology learning models further emphasize that what happens during initial encounters—whether use feels effortful, confusing, or failure-prone—strongly shapes whether a tool becomes part of everyday practice or is abandoned [4, 44]. Interpreted through this lens, our results suggest that recent gains may reflect expanded trial, while persistent barriers during learning and early use continue to limit sustained engagement—highlighting the need for support that extends beyond onboarding [43–45].

A key issue is that patient portals are feature-rich interfaces optimized for breadth, not for older adults’ age-related needs. Older adults often struggle to navigate feature-rich user interfaces, and this challenge is consistently documented as more pronounced than for younger users [24, 27, 53–55]. Reported reasons include difficulty recognizing navigation structures, reluctance to explore unfamiliar menus, and confusion between neighboring or visually similar icons and buttons [21, 24, 52]. These usability costs intensify when portal interfaces change. Because effective use depends on a stable sense of “how this portal works,” even modest redesigns can require older adults to form a new mental model. Mental models provide the organizing structure people use to interpret systems; extending an existing model typically requires fewer cognitive resources than acquiring a new one, and building a new model can place heavier demands on older adults’ working memory [15, 33].

Without continued support, updates can therefore erase hardwon familiarity. When scheduling, messaging, and test results are primarily routed through portals, recurring login, navigation, and comprehension problems can delay or disrupt routine care tasks—especially for older adults who need the most support. Addressing this gap requires investing in tech support during continued use [53], including “just-in-time” assistance (e.g., accessibility tools layered onto portals) and reliable human help in places older adults already turn to (clinics, libraries, senior services) [43, 45].

Such initiatives require strong evidence because what gets measured is often what gets funded, scaled, and justified in public policy. To align with theory distinguishing trial from acceptance/routine use, research should operationalize “portal use” as stage-specific rather than treating any one-time log-in as engagement. Measures should capture not only access, but whether older adults can complete meaningful tasks and recover from breakdowns (e.g., task success, comprehension, confidence, time-to-recovery, caregiver/proxy dynamics). This need is underscored by our very high heterogeneity, which likely reflects wide variation in study contexts and definitions and limits the precision of pooled estimates; more consistent operationalization and reporting would strengthen the evidence base for investing in continued patient portal use in older adults.

## Figures and Tables

**Figure 1:**
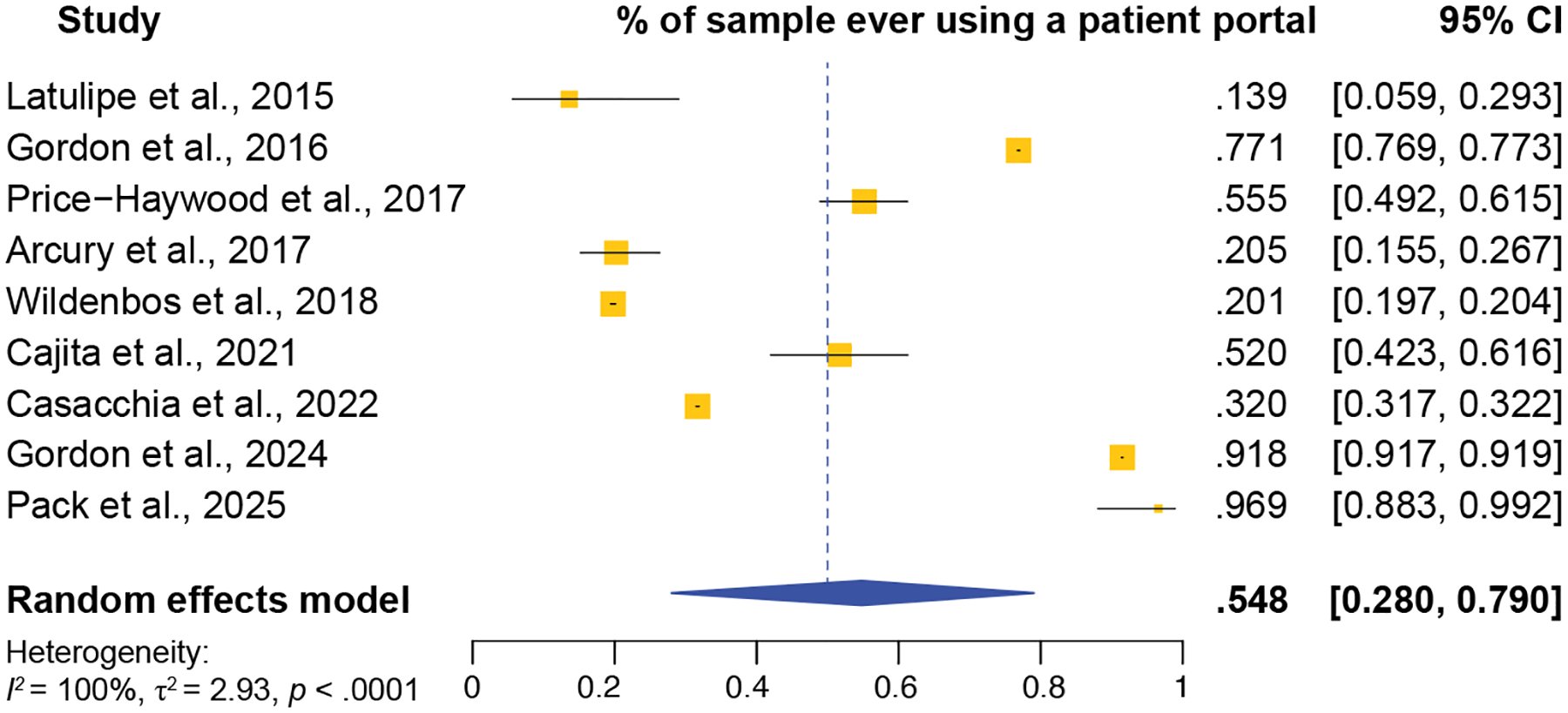
Forest plot of study-level prevalence estimates for older adults who have used a patient portal (at least once). Across *k* = 9 studies, the pooled prevalence was 0.55 (95% CI: 0.28–0.79), with very high heterogeneity—suggesting that, overall, about 55% of older adults had used a patient portal at least once, but rates varied substantially across studies.

**Figure 2:**
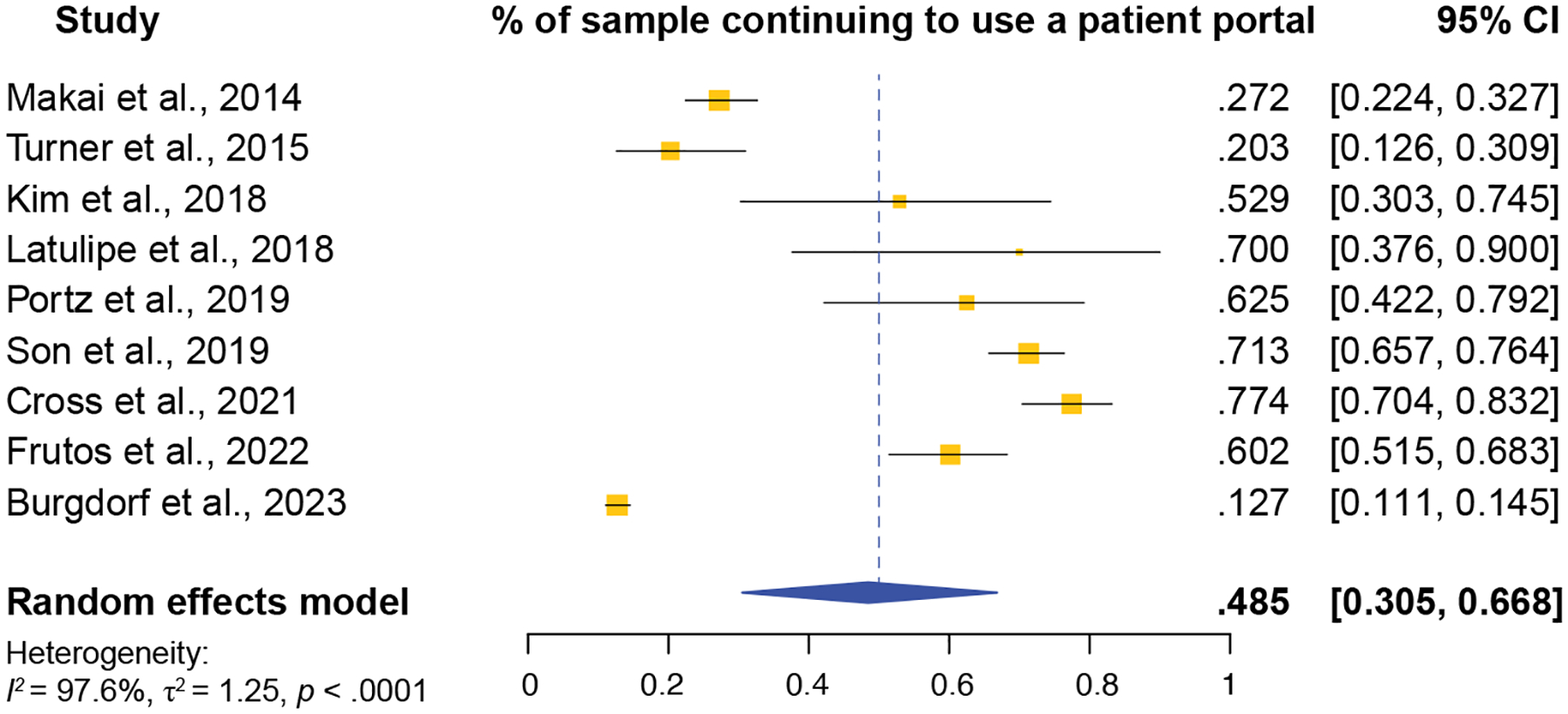
Forest plot of study-level prevalence estimates for older adults who are continuing users of a patient portal. Across *k* = 9 studies, the pooled prevalence was 0.49 (95% CI: 0.31–0.67), with very high heterogeneity—indicating that, overall, about 49% of older adults were continuing portal users, but rates varied substantially across studies.

**Figure 5:**
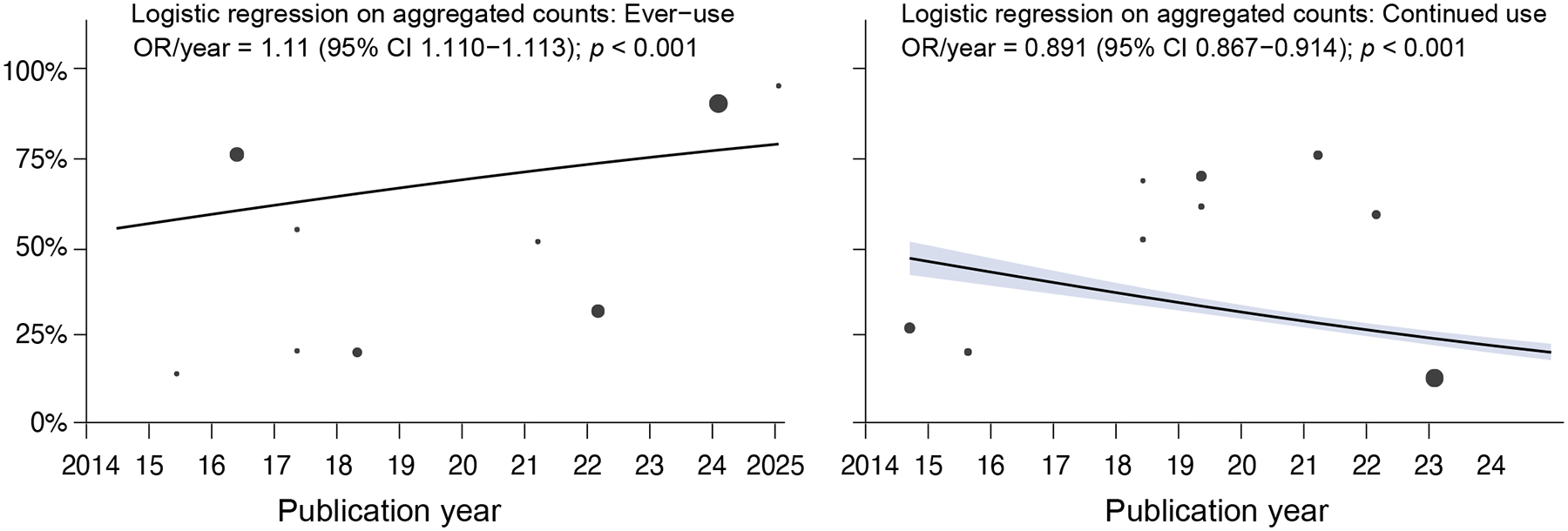
Logistic regression trend plots of patient portal engagement by publication year using aggregated counts (sample-size–weighted). The annual odds of older adults using a patient portal (at least once) increased over time (left; OR/year = 1.11), while the annual odds of continued use decreased (right; OR/year = 0.89). Thus, the odds of older adults ever using a patient portal were about 11% higher for each more recent year, whereas the odds of continued use were about 11% lower for each more recent year.

## CCS Concepts

• Social and professional topics → Seniors; • Human-centered computing → Human computer interaction (HCI); Empirical studies in HCI.

## A Search Summary and Keywords

**Table 1:** Domains and keywords we used to perform the systematic literature search. We included the ADRD domain because research often identifies older adults with Alzheimer’s Disease and Related Dementia (and their informal caregivers) by diagnosis.

Domain	Keywords
Older Adults	“older adults”, “elderly”, “seniors”, “geriatric”, “aging population”, “older people”, “older persons”
Caregivers	“informal caregiver*”, “family caregiver*”, “carer*”, “unpaid caregiver*”, “care partner*”, “spousal caregiver*”, “adult children caregiver*”, “non-professional caregiver*”, “relative*”, “spouse*”, “adult child*”, “grandchild*”, “young carer*”, “younger family”
Alzheimer’s Disease and Related Dementia (ADRD)	“people with dementia”, “persons with dementia”, “patients with dementia”, “people living with dementia”, “diagnosed with dementia”, “people with mild cognitive impairment”, “diagnosed with MCI”, “PwD”
Patient Portal	“patient portal”, “patient portals”, “eHealth”, “e-health”, “health record”
Method	“user study”, “experiment*”, “observation*”, “interview*”, “focus group*”, “usability”, “case study”, “field study”, “participatory design”, “diary study”, “think aloud”, “cognitive walkthrough”, “survey*”, “questionnaire*”, “log analysis”, “user experience”, “interaction design”, “shared use”, “proxy use”, “healthcare proxy”, “proxy access”

## B Risk of Bias Results

**Table 2:** Overall risk-of-bias ratings using the JBI prevalence tool [31].

Overall RoB	All	Ever-used	Use
Low	1	1 [50]	0
Moderate	5	3 [10, 16, 17]	2 [8, 46]
High	12	5 [3, 9, 22, 34, 37]	7 [12, 14, 20, 23, 29, 36, 47]
